# Bicuspid aortic valve aortopathy is characterized by embryonic epithelial to mesenchymal transition and endothelial instability

**DOI:** 10.1007/s00109-023-02316-5

**Published:** 2023-05-10

**Authors:** David Freiholtz, Otto Bergman, Karin Lång, Flore-Anne Poujade, Valentina Paloschi, Carl Granath, Jan H. N. Lindeman, Christian Olsson, Anders Franco-Cereceda, Per Eriksson, Hanna M. Björck

**Affiliations:** 1 Section of Cardiothoracic Surgery, Department of Molecular Medicine and Surgery, Karolinska Institutet, Stockholm, Sweden; 2 Division of Cardiovascular Medicine, Center for Molecular Medicine, Department of Medicine, Karolinska Institutet, Karolinska University Hospital, Stockholm Solna, Sweden; 3 Department of Vascular Surgery, Department of Surgery, Medical Center Leiden, Leiden University, Leiden, the Netherlands

**Keywords:** Bicuspid aortic valve, Ascending aneurysm, EMT, Endothelial instability

## Abstract

**Abstract:**

Bicuspid aortic valve (BAV) is the most common congenital heart malformation frequently associated with ascending aortic aneurysm (AscAA). Epithelial to mesenchymal transition (EMT) may play a role in BAV-associated AscAA. The aim of the study was to investigate the type of EMT associated with BAV aortopathy using patients with a tricuspid aortic valve (TAV) as a reference. The state of the endothelium was further evaluated. Aortic biopsies were taken from patients undergoing open-heart surgery. Aortic intima/media miRNA and gene expression was analyzed using Affymetrix human transcriptomic array. Histological staining assessed structure, localization, and protein expression. Migration/proliferation was assessed using ORIS migration assay. We show different EMT types associated with BAV and TAV AscAA. Specifically, in BAV-associated aortopathy, EMT genes related to endocardial cushion formation were enriched. Further, BAV vascular smooth muscle cells were less proliferative and migratory. In contrast, TAV aneurysmal aortas displayed a fibrotic EMT phenotype with medial degenerative insults. Further, non-dilated BAV aortas showed a lower miRNA-200c-associated endothelial basement membrane LAMC1 expression and lower CD31 expression, accompanied by increased endothelial permeability indicated by increased albumin infiltration. Embryonic EMT is a characteristic of BAV aortopathy, associated with endothelial instability and vascular permeability of the non-dilated aortic wall.

**Key messages:**

- Embryonic EMT is a feature of BAV-associated aortopathy.
- Endothelial integrity is compromised in BAV aortas prior to dilatation.
- Non-dilated BAV ascending aortas are more permeable than aortas of tricuspid aortic valve patients.

**Supplementary Information:**

The online version contains supplementary material available at 10.1007/s00109-023-02316-5.

## Introduction

Ascending aortic aneurysm (AscAA) is a silent, potentially life-threatening disease manifesting as a localized dilatation of the ascending aorta. Bicuspid aortic valve (BAV), the most common congenital heart defect with an estimated prevalence of ~ 1% [1, 2], has a strong association with AscAA with up to 50–70% of all BAV patients requiring ascending aortic surgery at some point during their lifetime [2, 3]. The mechanism behind BAV formation is unknown, but a defective epithelial-to-mesenchymal transition (EMT), and a faulty signaling between different cardiac progenitor cells thereof, during semilunar valve formation has been highlighted [4–6]. This link between EMT and BAV occurrence was recently also strengthened by a large genome-wide association study, in which a deleterious missense variant in the EMT-related gene *MUC4* was associated with BAV formation [7]. Mechanistical studies in zebrafish further showed that loss of MUC-4 led to a delay in cardiac valvular development, putting *MUC4* in the context of aortic valve malformation. Importantly for the context of this study, in pioneering experiments performed by Epstein et al., the faulty embryonic EMT signaling was further extended to link defective valve formation with postnatal aortic abnormalities, such as AscAA formation [6, 8–10]. In line with this, we and others have previously implicated EMT in association with aortic dilatation in patients with non-familial BAV [11–13], which represents the vast majority of BAV cases, although it does not show traditional signs of post-natal EMT, such as elastin fragmentation, extracellular matrix deposition, and smooth muscle cell loss [14, 15]. Instead, the dilated BAV aortic wall seems structurally well preserved, devoid of tissue resident fibroblasts, and clearly different from that of aneurysmal tricuspid aortic valve (TAV) patients [16, 17]. Additionally, key signs of inflammation and fibrosis are lacking in BAV-associated aneurysms [18], which may allude to the fact that another type of EMT is active in BAV adult aortas, possibly originating from embryonic development and the defective valve formation (i.e., embryonic EMT in contrast to fibrotic postnatal EMT).

The aim of this study was to investigate EMT in ascending aortas of patients with non-familial BAV from a subtype-specific perspective (embryonic vs. fibrotic EMT), as well as delineating the state of the endothelium, to further understand the pathology behind general BAV-associated aortic disease. We hypothesize that, in contrast to TAV AscAA, an embryonic-like EMT is active in BAV ascending aorta and contributes to aneurysm formation possibly by compromising the intimal/endothelial stability. An increased knowledge of the specific mechanism behind BAV aneurysm susceptibility is of importance to improve risk assessment, surveillance, and possible novel treatment targets for these patients**.**

## Material and methods

### Patients

Patients from the Advanced Study of Aortic Pathology (ASAP) and Disease of the Aortic Valve, Ascending Aorta and Coronary Arteries (DAVAACA) cohorts were studied. The cohorts have been described in detail elsewhere [19, 20]. In brief, all patients underwent elective open-heart surgery for aortic valve disease and/or ascending aortic aneurysm (AscAA) with or without coronary artery disease at the Karolinska University Hospital, Stockholm, Sweden. Tissue biopsies were collected from the anterior part of the ascending aorta, at the site of aortotomy a few centimeters above the aortic valve. Patients were classified according to aortic valve morphology (BAV or TAV) and aortic dilatation (non-dilated (ND) or dilated (D), where aortic diameters of > 45 mm were considered dilated and aortas < 40 mm were classified as non-dilated). Patients with a ND aorta underwent isolated aortic valve surgery, either replacement or repair, leaving the ascending aorta intact. Patients with syndromic aortic pathologies, dissection, or significant coronary artery disease according to angiography were omitted from analyses. The study was approved by the Human Research Ethics Approval Committee in Stockholm (application no. 2006/784–31/1 and 2012/1633–31/4). Written consent was obtained from all patients according to the declaration of Helsinki. In total, *n* = 131 BAV (*n* = 55 ND and *n* = 76 D) and *n* = 80 TAV (*n* = 41 ND and *n* = 39 D) patients were included. Patient characteristics are presented in Supplementary Tables S1, S2, and S3.

### Movat’s pentachrome staining

Tissue sections (BAV-D *n* = 11 and TAV-D *n* = 8) were deparaffinized, re-hydrated, and rinsed in distilled water. Then, sections were stained twice in 1% Alcian blue solution for 15–25 min, rinsed in running warm water until clear, and incubated in alkaline alcohol solution for 30 min. Following rinsing in running tap water, tissue sections were stained in elastic hematoxylin solution for 20 min, rinsed in running warm tap water, and differentiated in 2% aqueous 10% ferric chloride solution for minimum 5 s and up to 2 min. Then, slides were placed in 5% sodium thiosulfate solution for 1 min, washed in running tap water, rinsed in distilled water, and subsequently stained in Biebrich scarlet/acid fuchsin solution for 1–1.5 min. Following rinsing in distilled water, slides were rinsed in 1% acetic acid solution for 7–12 s, placed in 5% aqueous phosphotungstic acid solution for 7–12 min, and rinsed again in distilled water. Lastly, slides were rinsed in 1% acetic acid solution for 8–10 s, incubated in two changes of 100% ethanol, stained in 4% alcoholic saffron solution for 1.5 min, and finally, quickly rinsed in 100% ethanol before de-hydration. A detailed protocol of solutions can be found in [21].

### Cell culture

Human aortic smooth muscle cells (SMCs) were isolated from aneurysmal aorta of BAV (*n* = 5) and TAV (*n* = 3) patients, as described previously [22], and cultured in smooth muscle cell basal media with growth supplements, including 10% fetal calf serum and penicillin/streptomycin (PromoCell, C-22111). Cells were used in passage 6–7.

### Migration and proliferation assay

Cells were seeded onto 96-well plates coated with collagen I (OrisTM Cell Migration assay, Platypus technology CMACC1.101) at a density of 25,000 cells/well. After 16 h, ORIS plugs were removed, and cells were washed with PBS prior to incubation with CellTracker Green CFMDA dye (Life Technologies, C2925) for 45 min. After incubation, cells were washed once in PBS and 100 µl of culture medium was added to each well. The plate was subsequently placed into the IncuCyte live-cell analysis system (Sartorius) and scanned repeatedly for 24 h.

### Gene and miRNA expression analyses

The aortic intima-medial layer was separated manually from the adventitia, and mRNA was extracted from the intima-medial using RNeasy Mini kit (Qiagen, Crawley, UK) including treatment with RNase-free DNase set (Qiagen), according to manufacturer’s instructions. For matched miRNA and mRNA expression analysis, expression profiles were generated by Applied Biosystems Clariom D Array, and GeneChip miRNA 4.0, from in total 12 BAV-ND and 12 BAV-D patients. The quality of RNA was analyzed with an Agilent 2100 bioanalyzer (Agilent, Santa Clara, CA) and quantity was measured by a NanoDrop (Thermo Scientific, Waltham, MA). The mean RNA integrity number (RIN) was 7.1 ± 0.6. RNA samples with a RIN below 6 were excluded. The Affymetrix GeneChip® Human Exon 1.0 ST array and protocols were used for expression profiling, as previously described [18]. In total, gene expression was measured in 119 patients (31 BAV-ND, 44 BAV-D, 23 TAV-ND, and 21 TAV-D).

### Protein expression analyses

Global protein expression has previously been measured using HiRIEF and LC–MS/MS in the intima-media layer of 21 patients (5 BAV-ND, 5 TAV-ND, 6 BAV-D, and 5 TAV-D). In the present study, LAMC1 and TAGLN expression levels were evaluated in non-dilated samples. A detailed description of the LC–MS/MS method can be found in Maleki et al. [13].

### Functional enrichment analysis

The database we used for functional enrichment analysis was the Molecular Signatures Database (MSigDB, v5.0). Among MSigDB, we used the following two collections. h, hallmark gene sets; c5, GO biological processes as gene symbols. To eliminate redundancy and focus on the main research interest of this study, in c5, we selected terms that were related to EMT. Enrichment analysis was implemented using a hypergeometric test, by in-house R scripts, integrated with clusterProfiler R package [23].

### Immunohistochemistry

Aortic surgical biopsies were embedded in OCT, snap frozen, and sectioned to a thickness of 8 µm in a cryostat (CryoStar NX70). The sections were fixed in methanol for albumin, 4% formaldehyde for vWF and CD31, and acetone for laminin γ1, and blocked with 10% goat serum for 60 min for vWF and CD31, or with 5% goat serum (Vector laboratories) for 30 min for laminin γ1. Then, sections were subsequently incubated overnight at 4 °C with primary antibodies; anti-albumin antibody (ALB (F10) Santa Cruz sc-271605) diluted 1:100, vWF (DAKO A0082) diluted 1:2000, CD31 (Abcam ab9498) diluted 1:400, or laminin γ1 (2E8 Merck Millipore, MAB1920) (diluted 1:200). Slides were then incubated with an Alexa Fluor 568-conjugated goat anti-mouse secondary antibody (Invitrogen A11004), Alexa Fluor 647-conjugated goat anti-rabbit secondary antibody (Invitrogen A21244), or Alexa Fluor 647-conjugated goat anti-mouse secondary antibody (Invitrogen A21235) diluted 1:600 for 2 h in room temperature. Nuclei were counterstained with 4,6-diamidino-2-phenylindole (DAPI Sigma MBD0015) diluted 1:4000 or 1:6000 for laminin γ1. Stainings were mounted in Fluoromount-G (Invitrogen 00–4958-02). Tissue sections were then visualized with a laser scanning confocal microscope (Nikon) or a Pannoramic MIDI II slide scanner (× 20 objective, 3DHISTECH Kft., Budapest, Hungary). Albumin fluorescence signal area and intensity were quantified with Image Pro (Media Cybernetics). Laminin γ1 and CD31 fluorescence signal intensity were assessed in tissue sections displaying a continuous monolayer along the endothelium was quantified using ImageJ (Version 1.8.0). In total, between 5 and 11 patients were included in each patient group. The specific number of patients included in each antibody staining is indicated in the figure legends.

### Statistical analysis

Statistical analyses were performed using IBM statistics (v.27, Armonk, NY), Prism 9 and R. Differential gene expression was investigated using Student’s t-test assuming unequal variance. Differences in cell migration, proliferation, and fluorescence intensity were analyzed by Mann–Whitney U-test; data were expressed as mean ± standard error of the mean (SEM). *P* < 0.05 was considered statistically significant.

## Results

### Structural differences between BAV and TAV dilated ascending aortas

**Fig. 1:**
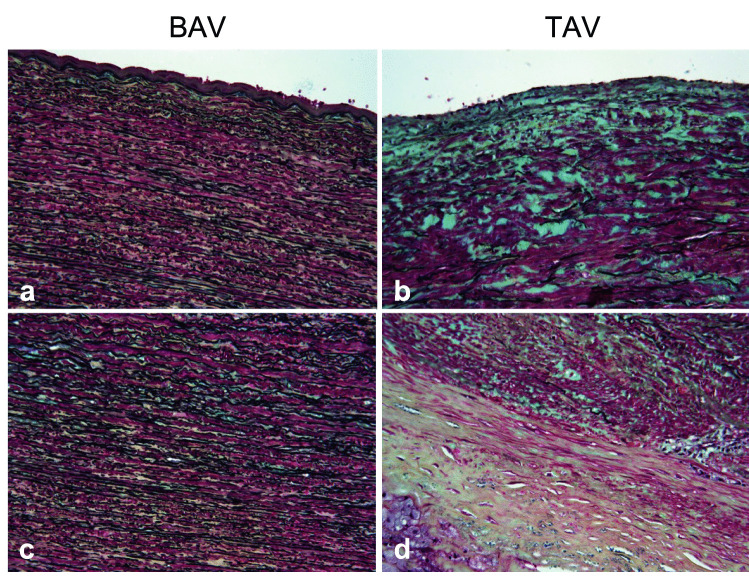
Movat pentachrome staining of dilated ascending aortas (magnification × 20) from patients with BAV (*n* = 11) and TAV (*n* = 8), respectively. BAV, bicuspid aortic valve; TAV, tricuspid aortic valve

Although signs of an ongoing EMT have been demonstrated in ascending aortas of patients with BAV, key characteristics of traditional postnatal EMT, such as fibrosis, inflammation, and excessive collagen production, appear to be lacking [16, 18]. To further evaluate EMT associated with BAV aortopathy, we performed histological staining of BAV-dilated ascending aortas using Movat’s pentachrome stain, using tissue specimens from TAV-associated AscAA as a reference. Tissue specimens were evaluated according to proteoglycans and collagen content and morphology, and integrity of elastic fibers. As demonstrated in Fig. 1, dilated aortas from patients with BAV showed high grade of structural organization with visually intact elastic fibers and low levels of proteoglycan and collagen. TAV aneurysmal aortas, on the other hand, were disorganized with evident elastin fragmentation and proteoglycan and collagen deposition, consistent with a fibrotic type of EMT.

### Increased migratory and proliferative potential of TAV aortic smooth muscle cells

**Fig. 2:**
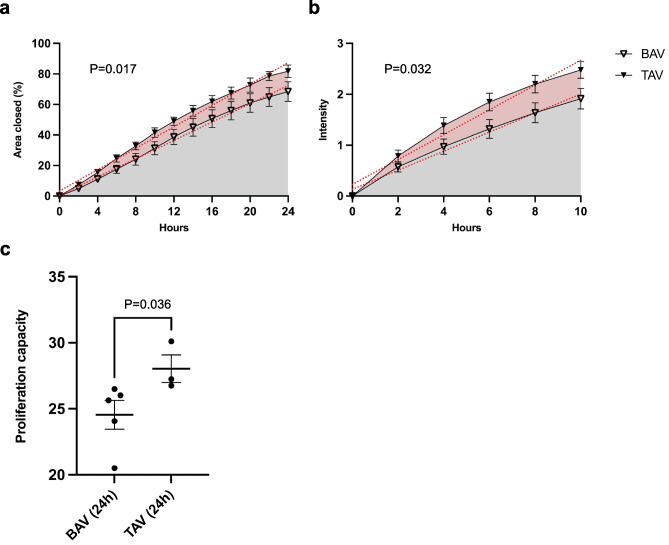
ORIS migration assay using ascending aortic vascular smooth muscle cells isolated from patients with BAV and TAV, respectively, visualized by IncuCyte live cell imaging. **a** Area covered by cells at 0–24 h of growth and **b** fluorescent dye intensity at 0–10 h of growth. Red dotted lines represent the regression line for BAV (*n* = 5) and TAV cells (*n* = 3), respectively. **c** proliferation at 24 h, *P* = 0.036, Mann Whitney U-test. BAV, bicuspid aortic valve; TAV, tricuspid aortic valve

In contrast to embryonic EMT, postnatal EMT manifesting in adult or mature tissue involves the formation of resident tissue fibroblasts with greater proliferative potential [15] or alterations in the endothelial phenotype, referred to as endothelial to mesenchymal transition. To assess potential differences in proliferation and migration between BAV and TAV cells, primary smooth muscle cells were isolated from BAV and TAV ascending aortas and an ORIS migration assay was performed during live cell imaging using the IncuCyte. A fluorescent dye was added to each sample to assess cell proliferation. As seen in Fig. 2a, b, TAV aortic SMCs migrate faster than BAV aortic SMCs, indicated by a greater area under the curve (AUC) (*P* = 0.017, comparing the slope of each simple regression line) and a higher fluorescence (*P* = 0.032). In addition, although covering a greater migration area at 24 h, TAV cells had the same signal intensity as BAV cells resulting in a higher area-to-signal ratio at 24 h (Fig. 2c) and thus a higher proliferation rate (*P* = 0.036).

### Genes related to endocardial EMT are enriched in BAV-associated aneurysm

To further disentangle whether different types of EMT may be active during BAV- and TAV-associated aortic dilatation, we performed a differential expression analysis to identify BAV- and TAV-specific dilatation genes (genes were classified according to changes in expression from non-dilated to dilated aortas in BAV and TAV patients, respectively). Then, a functional enrichment analysis was implemented on genes in each category (flowchart outlined in the Supplementary Fig.). Hallmark MT as well as EMT-related Gene Ontology (GO) terms from MSigDB were used in the functional enrichment analysis (selected GO terms presented in Supplementary Table S4). As shown in Table 1, TAV-specific genes were enriched for the most generic classification of EMT, i.e., Hallmark EMT (*P* = 0.011) but none of the more specific EMT-related GO terms. BAV-specific genes showed a stronger association with Hallmark EMT (*P* = 0.0048), as well as the GO-term EMT (*P* = 0.0046). Interestingly, BAV-specific genes also showed a borderline enrichment for EMT GO terms related to endocardial cushion formation (*P* = 0.063 and 0.069), Table 1. This suggests that different subtypes of EMT may be active during aortic dilatation in patients with BAV and TAV, and that embryonic EMT could be more important to process of dilatation in BAV patients.

**Table 1:** Enrichment or EMT-related GO terms in BAV- and TAV-specific dilatation genes

**BAV-specific dilatation genes**	
**EMT-related Gene Ontology (GO) terms**	***P*-value**
GO EMT	0.0046
HALLMARK EMT	0.0048
GO regulation of EMT	0.034
GO EMT involved in endocardial cushion formation	0.063
GO Positive regulation of EMT involved in endocardial cushion formation	0.069
Positive regulation of EMT	0.083
GO Regulation of EMT involved in endocardial cushion formation	0.098
GO regulation of cardiac EMT	0.20
GO negative regulation of EMT	0.31
-	
**TAV-specific dilatation genes**	
**EMT-related GO terms**	***P*-value**
HALLMARK EMT	0.011

*BAV *bicuspid aortic valve, *EM *epithelial to mesenchymal transition, *TAV *tricuspid aortic valve

### Different miRNAs are enriched for BAV- and TAV-specific EMT genes

**Fig. 3:**
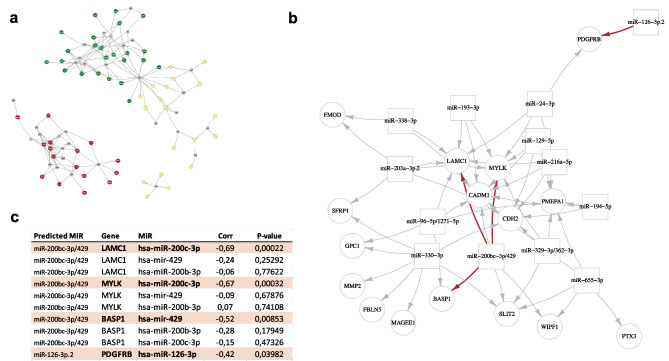
**a** Network of BAV-specific (red), TAV-specific (green), and common (yellow) Hallmark EMT genes and their predicted miRNAs (identified using TargetScan). Gray squares and arrows mark predicted miRNAs and their targets. **b** Zoomed network of BAV-specific Hallmark EMT genes and their predicted miRNAs. Red arrows show negative correlation between gene and miRNA expression with corresponding *P* and correlation values presented in **c**. BAV, bicuspid aortic valve; TAV, tricuspid aortic valve

MicroRNAs are important regulators of gene expression and have been implicated in the development of aneurysmal disease [24]. To investigate which miRNAs may play a role in the differential regulation of EMT genes changing with dilatation in BAV and TAV, a microRNA target analysis was performed. Genes classified according to the Hallmark EMT gene set were selected as input as this was the only EMT-term enriched in both BAV- and TAV-specific groups (Table 1). Interestingly, TargetScan and subsequent network analysis revealed that miRNAs predicted to target BAV- and TAV-specific EMT genes were specific for each group, with no overlap between BAV- and TAV-associated miRNAs (Fig. 3a).

### miR200 family is a master regulator of EMT-related BAV-specific dilatation genes

To investigate the regulation of the predicted miRNAs on BAV-specific target genes, matched miRNA and gene expression were measured in ascending aortic biopsies from patients with BAV. Significant (alpha 0.05) inverse miRNA-gene pair correlations are highlighted in red in the zoomed BAV network (Fig. 3b). This identifies miR-200c as a key regulator, specifically correlating with *LAMC1* (*P* = 0.00022, Pearson − 0.69) and *MYLK* (*P* = 0.00032, Pearson − 0.67) expression (Fig. 3c). Moreover, miR-429 was inversely correlated with *BASP1* expression (*P* = 0.0085, Pearson − 0.52).

### Decreased LAMC1 and CD31 expression in BAV AscAA

**Fig. 4:**
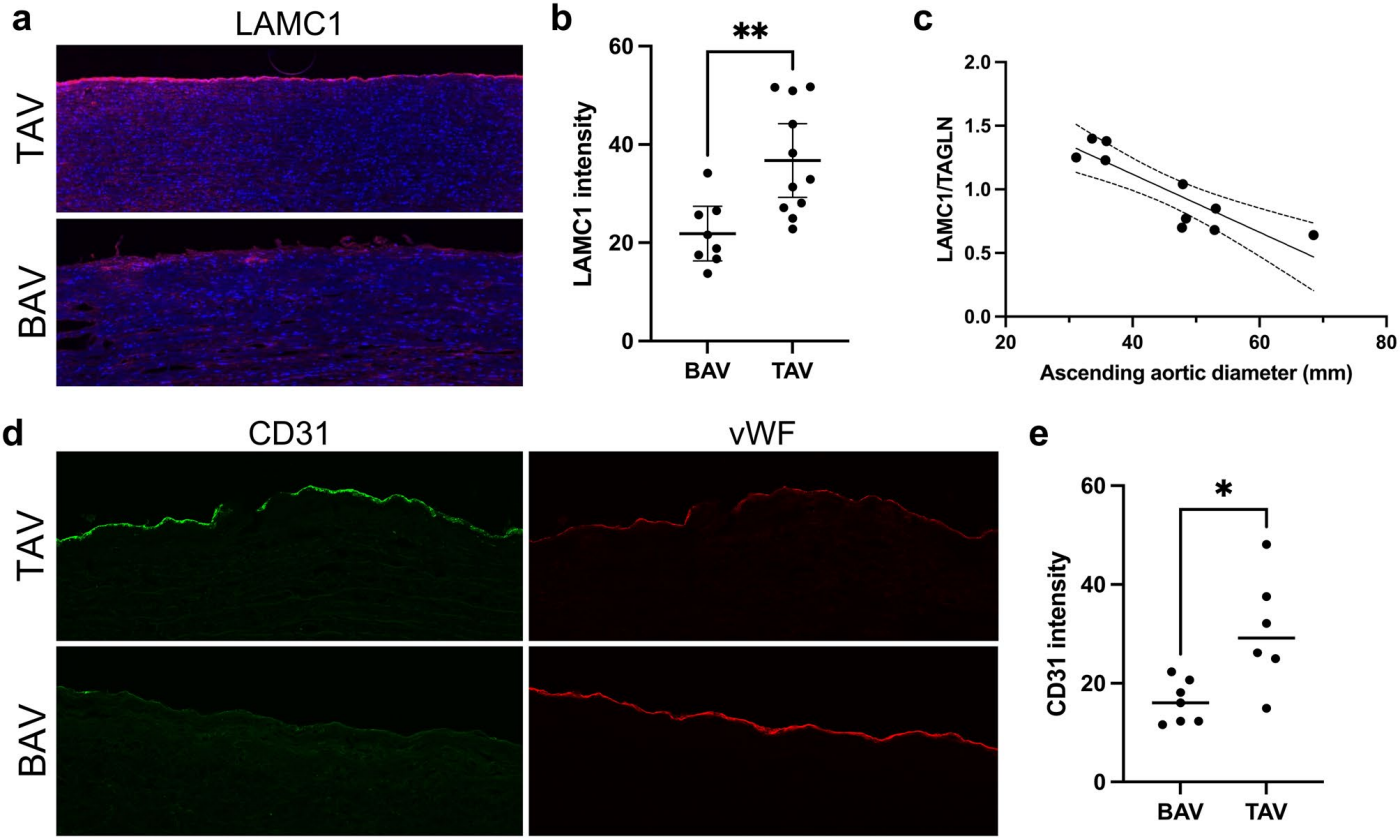
**a** Laminin γ1 (red) expression (magnification × 10) and **b** quantification, non-dilated ascending aortas of TAV (*n* = 11) and BAV (*n* = 8) patients. **c** LAMC1/TAGLN intima-media protein expression (global protein expression measured previously using HiRIEF LC–MS/MS, described previously in Maleki et al. [13]) in relation ascending aortic diameter in patients with BAV (*n* = 11), *P* = 0.004, Pearson correlation =  − 0.818. **d** CD31 (green) and vWF (red) expression (magnification × 10) and **e** quantification, non-dilated ascending aortas of BAV (*n* = 7) and TAV (*n* = 6) patients. BAV, bicuspid aortic valve; TAV, tricuspid aortic valve; D, dilated; ND, non-dilated

We have previously shown an up-regulation of miR-200c in the BAV non-dilated aorta compared with TAV [12]. To investigate whether the basement membrane (BM) laminin γ1 (encoded by *LAMC1*) expression is compromised in the BAV ascending aorta prior to dilatation, laminin γ1 protein expression was assessed using immunofluorescence. As can be seen in Fig. 4a, b, laminin γ1 expression was lower in the endothelial BM of BAV patients compared with TAV patients (*P* = 0.0036). Of note, analysis of previously acquired aortic intima-media proteomic expression [13] showed that LAMC1/TAGLN protein expression was inversely associated with ascending aortic dimensions in BAV patients (*P* = 0.004, Pearson correlation =  − 0.818) (Fig. 4c), suggestive of a BM reorganization during aortic dilatation. Note that, as the proteomic data represents the bulk intima-media expression, LAMC1 expression was normalized to the SMC protein TAGLN prior to analysis. To further assess intimal integrity, we stained for endothelial expression of CD31, a junctional protein tightly linked to endothelial barrier function and junction integrity [25]. Indeed, the expression of CD31 was markedly lower in BAV aortas compared with non-dilated TAV aortas (*P* = 0.014) (Fig. 4d, e).

### Increased endothelial permeability in BAV ascending aorta

**Fig. 5:**
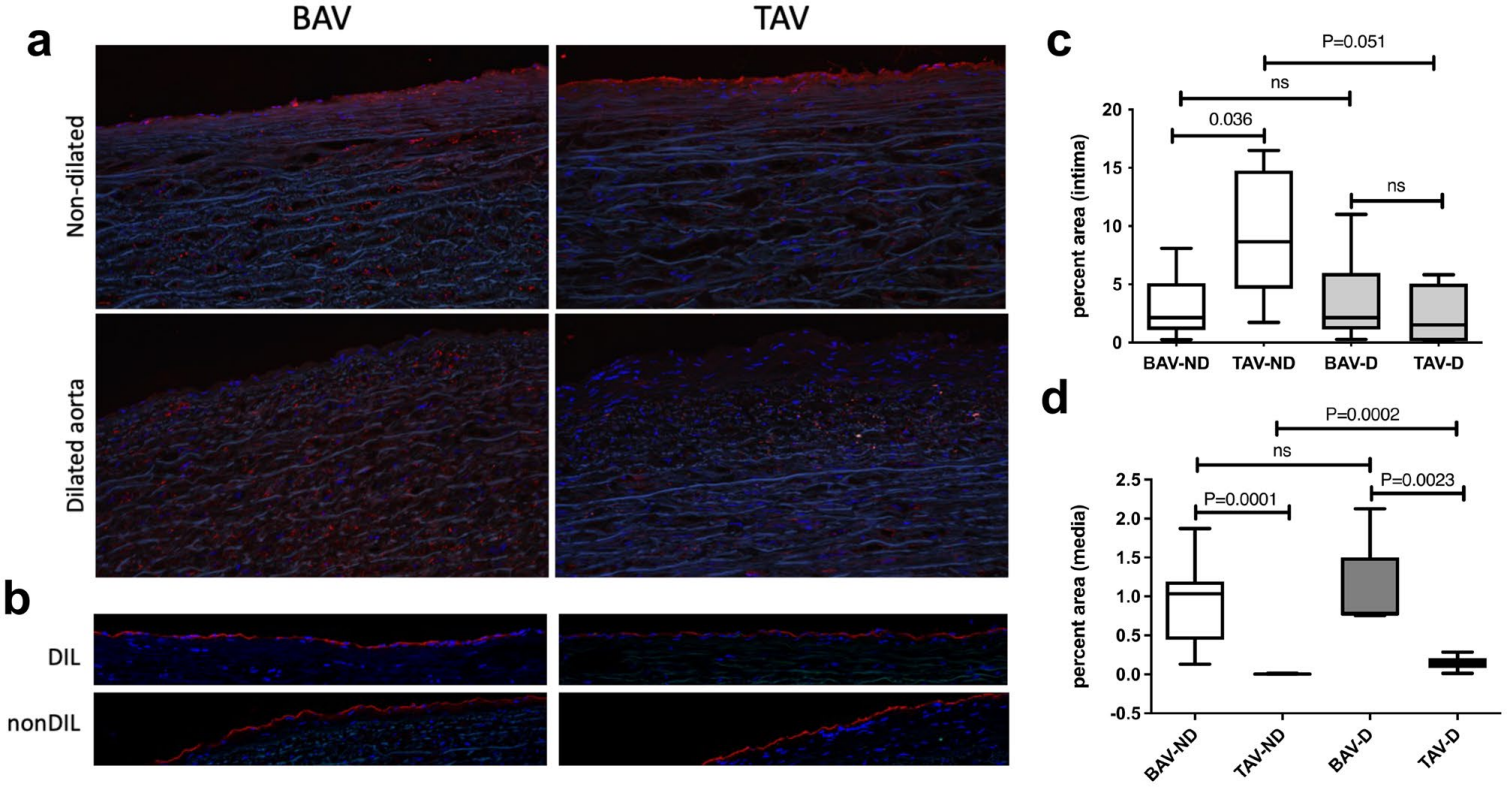
**a** Endothelial and medial albumin expression in non-dilated and dilated ascending aortas of BAV (*n* = 9 non-dilated, *n* = 5 dilated) and TAV (*n* = 9 non-dilated, *n* = 7 dilated) patients (magnification × 10). **b** vWF expression, non-dilated and dilated ascending aortas, BAV (*n* = 9 non-dilated, *n* = 5 dilated), and TAV (*n* = 9 non-dilated, *n* = 7 dilated) patients (magnification × 10). **c**, **d** Quantification of albumin staining in intima and media layers, respectively. BAV, bicuspid aortic valve; TAV, tricuspid aortic valve; vWF, von Willebrand factor

To assess whether the decreased LAMC1 and CD31 expression is associated with any functional alterations of the endothelial layer, albumin infiltration was evaluated as an indicator of endothelial permeability in tissue sections from non-dilated BAV and TAV aortas. As can be seen in Fig. 5a, c, the non-dilated TAV endothelium displayed increased endothelial staining of albumin compared to the non-dilated BAV endothelium, implying a more intact vascular endothelium in TAV patients (*P* = 0.036). This was accompanied by less albumin infiltration in the media of TAV patients, in both non-dilated and dilated states (*P* = 0.0001 and *P* = 0.0023 for non-dilated and dilated aortas, respectively, Fig. 5a, d). Notably, as dilatation occurs in patients with TAV, the endothelium likely becomes compromised and loses integrity, as indicated by less endothelial albumin staining in dilated TAV (Fig. 5a, d, P = 0.051). There was no difference in endothelial albumin staining between nondilated and dilated BAV (Fig. 5a, c). Importantly, concomitant staining for vWF showed the presence of endothelial cells in all sections (Fig. 5b). Together these observations suggest that vascular integrity, and specifically endothelial integrity, is disrupted in non-familial BAV already prior to dilatation, which is also indicated by a higher albumin staining in BAV non-dilated media compared with TAV (*P* = 0.0001).

## Discussion

The aneurysmal process is often associated with fibrotic changes of the ascending aortic wall and elastin fragmentation. However, in patients with a BAV, the dilated ascending aorta does not show clear signs of medial degeneration, but instead, evidence of compromised endothelial integrity has been put forth. Specifically, a process called EMT has been reported and suggested to contribute to the dilatation process [6, 10–13]. In the present study, the EMT process associated with BAV aortopathy was studied in-depth, and we report support of an EMT resembling that of embryonic origin being active in the ascending aorta of patients with non-familial BAV. Further, the embryonic EMT signature was associated with endothelial instability and aortic endothelial permeability, which was present already prior to aortic dilatation. We further show that EMT-related genes are regulated by unique miRNAs in BAV and TAV patients, respectively, suggestive of different types of EMT being active during aneurysm formation in the two patient groups.

The association between BAV and AscAA has been suggested to originate from embryogenesis and the spatiotemporally related processes of aortic valve formation and development of the primitive ascending aorta [9]. EMT plays a crucial role during both these processes, and a proper EMT signaling between cardiac progenitor cells and neural crest cells, which later migrates and contributes to the formation of the ascending aorta, is essential for normal aortic valve and ascending aortic development [8, 9, 26, 27]. Indeed, several studies in animal models suggest that aortic wall anomalies appear concomitantly with valve malformations through alterations of EMT-related genes and pathways [28, 29]. Additionally, variants in the EMT-related *ROBO4* gene have been identified in two probands with BAV and AscAA [10]. Here we undertook an ontology-based approach to disentangle which type of EMT is active in the ascending aorta of non-familial-type BAV patients and shows that the EMT-genes that are differentially expressed between non-dilated and dilated ascending aortas in BAV patients were related to the type of EMT involved in endocardial cushion formation and the regulation thereof. Although EMT in BAV aortas could be induced by perturbed flow caused by the malformed aortic valve itself [30, 31], its relation to endocardial cushion formation is noteworthy in the abovementioned context and further highlights a potential link to embryonic development and embryonic EMT specifically. Moreover, the differentiation of EMT types between BAV and TAV aortas was further strengthened by the observations that dilated BAV aortic specimens show a remarkable high degree of medial integrity and elastin organization, which is atypic for postnatal fibrotic EMT, as well as by the lower migratory and proliferative potential of VSMC isolated from BAV-ascending aortas compared with TAV VSMCs. In addition, it was further noted that the BAV-specific EMT genes were regulated by a completely different set of miRNAs than TAV-specific EMT genes, suggesting that EMT seen in BAV aortas is distinct from that observed in TAV aortas. Specifically, miR-200c was found to regulate BAV-specific but not TAV-associated EMT genes, which is in line with previous observations by us showing an up-regulation of miR-200c in the BAV endothelium compared with TAV [12].

To further elucidate the potential regulatory network associated with increased miR-200 expression, we performed a gene-miRNA interaction analysis including genes differentially expressed with aortic dilatation in BAV. Interestingly, miR-200c was significantly inversely correlated with the BM component LAMC1. Laminins are the major non-collagenous components of the basement membrane and have been implicated in a range of biological processes, including cell adhesion, differentiation, and migration. In large blood vessels, laminin subunit γ1 joins with laminin subunit β1 or β2, and subunit α4 or α5 to express laminin isoforms 411, 421, 511, or 521, respectively [32, 33]. A decrease expression of laminin γ1 chain, which was indicated by immunofluorescent staining in the BAV non-dilated aorta, implies a general decrease of laminin in the BM and/or an increased BM turnover and disassembly. It has previously been shown that knockout of laminin γ1 in mouse embryoid bodies prevents BM formation and results in failure of ectoderm epithelialization and an acceleration of mesoderm differentiation [34]. Moreover, laminins are known to regulate endothelial cell function and vascular permeability and may also regulate EMT [32, 35]. It may thus be speculated that a lower laminin content in the BAV aortic endothelial basement membrane may adversely affect the integrity of the intimal layer and possibly promote EMT. Similarly, a decreased expression of the endothelial junction protein CD31 (also known as PECAM1) is likely to attenuate endothelial barrier function, possibly by promoting EMT [25]. Indeed, we have previously shown clear signs of endothelial instability in association with the EMT-phenotype in BAV [6, 12, 13]. Furthermore, *BASP1* and *MYLK* expression was significantly inversely correlated with MiR-429 and MiR-200c expression, respectively, in BAV ascending aortas. It has previously been shown that transient *BASP1* knockdown by siRNA attenuates endothelial migration, and that homozygous *basp1* null zebrafish embryos display major vascular defects and upregulation of β-catenin-independent (non-canonical) Wnt signaling [36]. MYLK is one of the major RHOA kinases known to regulate endothelial cell invasion and migration during endocardial cushion tissue formation [37] and has previously been implicated in BAV aortopathy [11, 13].

To further elucidate the state of the BAV intima and the possible consequence of endothelial junction instability in relation to vascular permeability, we stained for the presence of plasma albumin in the aortic intima-media. Interestingly, we could see a significant increased albumin infiltration in both the non-dilated and dilated BAV aortas when compared with TAV counterparts. The non-dilated TAV endothelium displayed increased endothelial staining of albumin compared to the non-dilated BAV endothelium, implying a more intact vascular endothelium in TAV patients. Of note, concomitant staining for vWF showed the presence of endothelial cells in all sections. An increased vascular permeability and medial albumin infiltration have previously been reported in BAV probands with AscAA carrying a variant in the gene encoding ROBO4 [10]. Here we show that vascular instability and permeability seem to be a general feature of the BAV ascending aorta and present already prior to ascending aortic dilatation. Of note, these results are in line with a previous report investigating fetal gene expression that implicated endothelial cells as a major cell source contributing to BAV pathology [7]. Additionally, the potential link between BAV aortopathy and intimal instability is also supported by histological studies of the developing aorta, showing that BAV individuals display with intimal thickening prenatally with the intima becoming significantly thinner after birth [38]. Subsequently, the maturation of the BAV ascending aortic wall after birth is characterized by intimal remodeling and/or degeneration, likely contributing to vascular weakening and increased aneurysmal susceptibility.

### Limitations

The present study holds some limitations. Firstly, the cohort is a surgical cohort of adult BAV and TAV individuals selected for elective surgery, meaning that the patients included may be at different stages of their disease. This may introduce variability to the results. Secondly, due to its cross-sectional nature, results are mostly descriptive and cannot infer causality.

## Conclusions

In the present study, we propose embryonic EMT as a characteristic of BAV ascending aortopathy, in connection with endothelial basement membrane instability and permeability. In TAV aortas, on the other hand, fibrotic EMT dominates. During embryonic development, endothelial cells progress through several different fate transitions until they reach their specialized, highly differentiated adult state. It may thus be speculated that, as a consequence of defective EMT signaling during aortic valve and ascending aortic formation, BAV endothelial progenitor cells (that later populate and form the ascending aorta) fail to terminally differentiate and halt in a partially primordial, non-specialized state, thereby contributing to aneurysm susceptibility. Functional studies to support this hypothesis are thus needed.

## Supplementary Information

Below is the link to the electronic supplementary material.

Supplementary file1 (PDF 1569 KB)

## Data Availability

The datasets used and/or analysed during the current study are available from the corresponding author on request.

## List of Abbreviations

AscAA: Ascending aortic aneurysm
AUC: Area under curve
BM: Basement membrane
BAV: Bicuspid aortic valve
D: Dilated
EMT: Epithelial to mesenchymal transition
GO: Gene ontology
ND: Non-dilated
TAV: Tricuspid aortic valve
VSMC: Vascular smooth muscle cell
