# Supplementary material for: Bicuspid aortic valve aortopathy is characterized by embryonic epithelial to mesenchymal transition and endothelial instability
Source: J Mol Med (Berl). 2023 May 10;101(7):801–11. doi: 10.1007/s00109-023-02316-5 (PMC10299957; doi:10.1007/s00109-023-02316-5)

Supplementary materials

Bicuspid aortic valve aortopathy is characterized by embryonic epithelial to mesenchymal transition and endothelial instability

Short title: EMT and BAV ascending aortic aneurysm

David Freiholtz*, Otto Bergman*, Karin Lång, Flore-Anne Poujade, Valentina Paloschi, Carl Granath, Jan H N Lindeman, Christian Olsson, Anders Franco-Cereceda, Per Eriksson, Hanna M Björck

**contributed equally*

Page Content

Page	Content
2	Supplementary Table S1, Patient characteristics, migration/proliferation analysis
3	Supplementary Table S2, Patient characteristics, immunohistochemical stainings
4	Supplementary Table S3, Patient characteristics, gene expression analyses
5	Supplementary Table S4, Selected gene ontology terms related to EMT
5	Supplementary Figure, Flowchart, ontology analysis

Supplementary Table S1. Patient characteristics, BAV- and TAV-patients with dilated aortas from which vascular smooth muscle cells were isolated for migration and proliferation assays.

	BAV	TAV
**N**	5	3
**Age, years**	47.8 (2.3)	66.7 (9.4)
**Male sex**	5 (100)	3 (100)
**BSA, m^2**	2.1 (0.17)	2.02 (0.24)
**Triglycerides, mmol/L**	1.48 (0.46)	1.35 (0.64)
**Cholesterol, mmol/L**	4.78 (0.89)	3.1 (0.14)
**hsCRP, mg/L**	0.84 (0.4)	0.39 (0.13)
**Diabetes**	0 (0)	0 (0)
**Hypertension**	0 (0)	2 (67)
**Chronic inflammation**	1 (20)	0 (0)
**Vascular disease**	1 (20)	0 (0)
**Current smoker**	0 (0)	0 (0)
**Aortic valve and anerusym**		
**Aortic valve stenosis**	2 (40)	0 (0)
**Aortic valve regurgitation**	2 (40)	2 (67)
**Max ascending aortic diameter, mm**	31.8 (2.8)	37.6 (5.4)

BAV, bicuspid aortic valve; BSA, body surface area; hsCRP, high-sensitive C-reactive protein; TAV, tricuspid aortic valve. Continuous variables are presented as mean (SD) and ordinal variables are presented as n (%). Dilated (D) ascending aorta >45mm.

Supplementary Table S2. Patient characteristics, BAV and TAV patients included in immuno-histochemical stainings.

	BAV-ND	BAV-D	TAV-ND	TAV-D
**N**	12	15	18	15
**Age, years**	59.4 (11.6)	57.9 (11.3)	69.9 (9.7)	61 (12.5)
**Male sex**	10 (83)	10 (83)	13 (72)	13 (87)
**BSA, m^2**	2.00 (0.26)	2.00 (0.22)	1.96 (0.19)	2.01 (0.20)
**Triglycerides, mmol/L**	1.23 (0.54)	1.42 (0.89)	1.16 (0.37)	1.04 (0.42)
**Cholesterol, mmol/L**	4.21 (0.92)	5.25 (1.47)	4.51 (1.25)	4.49 (1.08)
**hsCRP, mg/L**	2.36 (2.44)	2.68 (2.57)	5.69 (12.9)	7.00 (20.1)
**Diabetes**	7 (58)	2 (13)	4 (22)	0 (0)
**Hypertension**	7 (58)	4 (27)	8 (44)	11 (73)
**Chronic inflammation**	0 (0)	1 (7)	0 (0)	1 (7)
**Vascular disease**	1 (8)	1 (7)	1 (6)	1 (7)
**Current smoker**	2 (17)	2(13)	1 (6)	0 (0)
**Aortic valve and anerusym**				
**Aortic valve stenosis**	7 (58)	8 (53)	11 (61)	0 (0)
**Aortic valve regurgitation**	6 (50)	7 (47)	8 (44)	10 (67)
**Max ascending aortic diameter, mm**	32.4 (5.82)	50.8 (5.14)	31.5 (5.27)	53.9 (6.49)

BAV, bicuspid aortic valve; BSA, body surface area; hsCRP, high-sensitive C-reactive protein; TAV, tricuspid aortic valve. Continuous variables are presented as mean (SD) and ordinal variables are presented as n (%). Aortic diameters of <40 mm were classified as non-dilated (ND) and aortic diameters of >45mm were considered dilated (D).

Supplementary Table S3. Patient characteristics, BAV and TAV patients included in the gene expression analyses.

	BAV-ND	TAV-ND	BAV-D	TAV-D
**N**	31	23	44	21
**Age, years**	58 (10)	67 (12)	61 (11)	63 (14)
**Male sex**	25 (81)	17 (74)	33 (75)	12 (57)
**BSA, m^2**	1.99 (0.24)	1.96 (0.19)	2.01 (0.21)	1.98 (0.25)
**Triglycerides, mmol/L**	1.28 (0.55)	1.03 (0.41)	1.56 (2.00)	1.19 (0.65)
**Cholesterol, mmol/L**	5.07 (1.10)	5.33 (0.85)	5.08 (1.29)	4.83 (1.06)
**hsCRP, mg/L**	2.58 (3.89)	4.04 (4.08)	2.22 (2.80)	6.77 (16.89)
**Diabetes**	4 (31)	0 (0)	3 (7)	0
**Hypertension**	14 (45)	12 (52)	21 (48)	12 (57)
**Chronic inflammation**	1 (3)	2 (8)	1 (2)	1 (4)
**Vascular disease**	0 (0)	0 (0)	0 (0)	0 (0)
**Current smoker**	4 (13)	2 (9)	5 (11)	2 (10)
**Aortic valve and anerusym**				
**Aortic valve stenosis**	19 (21)	13 (57)	30 (68)	0 (0)
**Aortic valve regurgitation**	11 (35)	10 (43)	11 (25)	9 (64)
**Max ascending aortic diameter, mm**	35.4 (3.4)	32.1 (4.3)	50.1 (3.2)	54.6 (6.1)

BAV, bicuspid aortic valve; BSA, body surface area; hsCRP, high-sensitive C-reactive protein; TAV, tricuspid aortic valve. Continuous variables are presented as mean (SD) and ordinal variables are presented as n (%). Aortic diameters of <40 mm were classified as non-dilated (ND) and aortic diameters of >45mm were considered dilated (D).

Supplementary Table S4. Selected gene ontology terms related to Epithelial to Mesenchymal Transition (EMT)

**EMT-related gene ontology terms**
Epithelial to Mesenchymal Transition (EMT)
Regulation of EMT, negative regulation of EMT
Positive regulation of EMT
cardiac EMT
Regulation of cardiac EMT
EMT involved in endocardial cushion formation
Regulation of EMT involved in endocardial cushion formation
Positive regulation of EMT involved in endocardial cushion formation

Supplementary Figure. Flowchart ontology analysis. Enrichment analysis of differentially expressed genes in BAV and TAV aortic intima-media, respectively. Gene ontology terms and Hallmarks were selected from the molecular Signatures Database (MSigDB, v5.0).

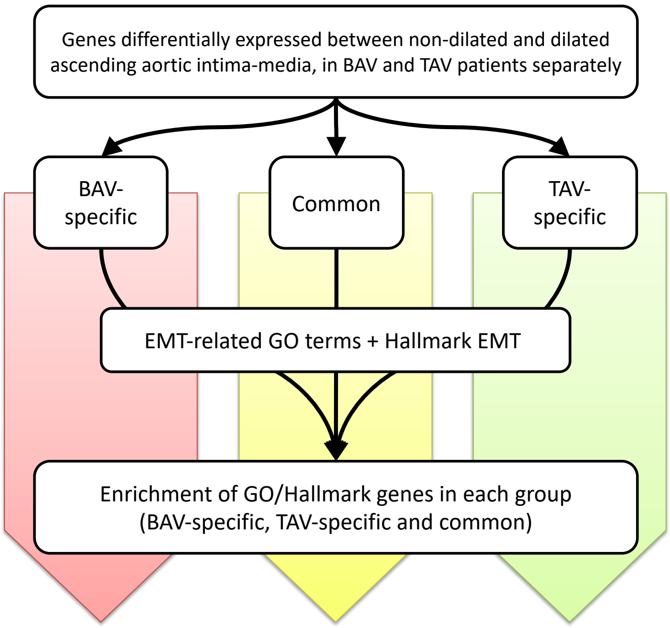

Supplement: Supplementary file 1 — Supplementary file1 (PDF 1569 KB) [file 109_2023_2316_MOESM1_ESM.pdf]
